# Co-occurrence of Cognitive Dysfunction and Depressive Disorders in Hemodialysis Patients: Demographic Patterns and Unmet Diagnostic Needs

**DOI:** 10.7759/cureus.99003

**Published:** 2025-12-11

**Authors:** Shikha Gautam, U.V. Kiran

**Affiliations:** 1 Human Development and Family Studies, Babasaheb Bhimrao Ambedkar University, Lucknow, IND

**Keywords:** chronic kidney disease (ckd), cognitive function, depressive disorders, hemodialysis, life orientation (optimism–pessimism), patient health status

## Abstract

Cognitive impairment and depressive disorders are common yet under-recognized comorbidities among patients with chronic kidney disease (CKD) undergoing dialysis. Moreover, life orientation, ranging from optimism to pessimism, plays a substantive role in modulating both clinical and psychosocial outcomes. This pilot cross-sectional study, conducted in Lucknow, India, assessed cognitive function, psychological outlook, and depressive symptoms among patients undergoing hemodialysis using the Montreal Cognitive Assessment (MoCA), the Life Orientation Test-Revised (LOT-R), and the Patient Health Questionnaire-9 (PHQ-9). Results indicated that 41% had mild cognitive impairment (MCI) and 10% had moderate cognitive impairment. Depression was widely observed in the sample, with 58% of patients presenting moderate symptoms and 7% experiencing moderately severe depressive levels. A pessimistic life orientation was reported by 32% of participants, while only 6% demonstrated strong optimism. Individuals aged 46-60 years and male patients showed a disproportionately higher level of cognitive and psychological difficulties. Additionally, comorbid diabetes (53%) and hypertension (67%) were significantly linked to adverse clinical and psychosocial outcomes. These findings align with global literature reporting a cognitive impairment prevalence of up to 80% in dialysis populations and high depression rates ranging from 13% to 76%. Despite their high prevalence, these conditions remain underdiagnosed in nephrology practice. The study underscores the need for routine cognitive and psychological assessment in dialysis units and early psychosocial interventions and informs the design of larger multicenter studies. Integrating such care could improve patient adherence, safety, and overall quality of life among patients with CKD.

## Introduction

Chronic kidney disease (CKD) has emerged as one of the most significant public health challenges of the 21st century. Globally, CKD affects more than 850 million people and ranks as one of the leading causes of death, according to the Global Burden of Disease 2019 study [1]. The prevalence is estimated to be around 9.1% worldwide, with a steady increase projected due to the rising incidence of diabetes, hypertension, and aging populations [2]. In India, the situation is particularly concerning, with prevalence estimates ranging from 10% to 17% in different cohorts [3]. The Indian CKD registry highlights an alarming surge in patients requiring renal replacement therapies such as hemodialysis, which continues to grow annually by nearly 15%-20% [4]. While the physical consequences of CKD, including metabolic imbalance, fluid overload, and cardiovascular complications, are well documented, the disease also exerts a profound psychological and cognitive toll [5]. These less visible dimensions often remain neglected in routine clinical care, despite their significant influence on patient outcomes, quality of life, and healthcare utilization.

Cognitive dysfunction is one of the most frequently reported complications in patients with CKD, particularly those undergoing dialysis. It encompasses deficits in memory, executive function, attention, and information processing, which may range from mild impairment to dementia-like presentations. Studies suggest that between 30% and 80% of dialysis patients experience some degree of cognitive impairment, depending on the tools used and the populations studied [6]. Multiple mechanisms underlie this phenomenon. Uremic toxins such as indoxyl sulfate and p-cresyl sulfate cross the blood-brain barrier, altering neurotransmission and promoting neuronal injury [7]. Vascular damage, driven by persistent hypertension and diabetes, contributes to cerebral small vessel disease and microinfarcts [8]. Chronic inflammation and oxidative stress, both hallmarks of CKD, accelerate neuronal degeneration. Furthermore, dialysis itself produces hemodynamic fluctuations that can lead to cerebral hypoperfusion and cumulative brain injury. Clinically, these impairments compromise treatment adherence, decision-making, and self-care [9]. They additionally predispose patients to more frequent hospitalizations, impair functional self-sufficiency, and adversely affect survival outcomes. Despite their prevalence and clinical impact, cognitive assessments are rarely incorporated into nephrology practice, leaving many patients undiagnosed until the deficits are advanced [10].

Equally concerning is the high prevalence of depressive disorders among patients with CKD. Depression, ranging from dysthymia to major depressive disorder, has been consistently identified as one of the most common psychiatric comorbidities in this population. Meta-analyses estimate that 20%-40% of dialysis patients experience moderate to severe depressive symptoms [11]. The relationship between CKD and depression is bidirectional. Physiologically, metabolic disturbances, hormonal changes, sleep disorders, and chronic fatigue predispose patients to mood disturbances [12]. Psychologically, the ongoing demands of lifelong dialysis, stringent dietary limitations, and fear of mortality contribute to substantial emotional distress, which is further intensified by the financial burden of treatment, particularly in low- and middle-income settings such as India, where dialysis often incurs significant out-of-pocket expenses, thereby exacerbating depressive symptoms [13].

Depression in CKD is not a benign accompaniment; it independently predicts poorer treatment adherence, increased cardiovascular risk, and higher mortality [14]. Moreover, the overlap between depressive symptoms and CKD manifestations such as fatigue, anorexia, and sleep disruption contributes to frequent underdiagnosis in clinical practice. Life orientation, reflecting the spectrum between optimism and pessimism, is another psychosocial construct relevant to chronic illness yet rarely studied in CKD populations. Optimism has been consistently associated with improved coping, resilience, and even lower mortality across diverse chronic conditions, including cardiovascular disease, cancer, and diabetes [15].

Optimistic patients are more likely to adhere to treatment regimens, utilize adaptive coping strategies, and maintain better mental health. Conversely, pessimism is linked with maladaptive coping, heightened stress, and vulnerability to depression and anxiety. Although studies in other chronic diseases have demonstrated the prognostic importance of optimism and pessimism, this construct has received little attention in nephrology. For patients undergoing dialysis, a treatment regimen that requires lifelong adherence, psychological endurance, and social support, life orientation may serve as a critical determinant of overall well-being [16].

The interplay between cognitive dysfunction, depressive symptoms, and life orientation is both complex and clinically significant. Cognitive decline can exacerbate feelings of helplessness, contributing to depression, while depression itself can impair cognitive performance and mimic dementia, a phenomenon known as pseudodementia [17]. Life orientation can act as either a buffer or a risk factor within this relationship. Optimistic outlooks may mitigate the psychological impact of cognitive decline and support resilience, while pessimistic orientations may amplify vulnerability, fostering a downward spiral of worsening cognition and mood [18]. Examining these domains collectively offers a more comprehensive perspective on the psychological well-being of individuals undergoing dialysis, shifting the focus beyond conventional disease-centric approaches to CKD care. Although the psychosocial burden of CKD is increasingly acknowledged, substantial gaps persist in both evidence and clinical practice. Cognitive impairment among dialysis patients frequently goes unrecognized, as mild or early deficits are seldom identified in the absence of systematic cognitive screening [19]. Depression is similarly overlooked, with symptoms misattributed to CKD itself rather than recognized as a separate treatable condition. Life orientation is almost entirely absent from clinical assessments, despite its established predictive role in other chronic illnesses. Furthermore, most studies addressing cognition and depression in CKD populations have been conducted in high-income countries, with limited representation of Indian patients who face unique cultural, socioeconomic, and healthcare challenges [20]. Research adopting an integrated approach to evaluating cognitive function, depressive symptoms, and life orientation remains scarce. Consequently, the broader spectrum of psychological vulnerability in CKD populations is inadequately captured, limiting opportunities for early identification and intervention.

The present study was therefore undertaken with three key objectives: firstly, to determine the prevalence of cognitive dysfunction, depressive disorders, and life orientation types among dialysis patients; secondly, to explore demographic variations in these domains, including age, gender, socioeconomic status, and education; and thirdly, to identify diagnostic gaps in the recognition of cognitive and emotional impairments within nephrology practice. Based on prior evidence, we hypothesized that cognitive dysfunction, pessimistic life orientation, and depressive symptoms would co-occur at high rates in the study population, with demographic variations influencing these outcomes and diagnostic gaps limiting timely recognition. This investigation, by integrating cognitive, emotional, and psychosocial dimensions, aims to advance the understanding of psychological vulnerability in CKD and provide a foundation for more comprehensive patient care.

## Materials and methods

Study design and setting

This research was designed as a cross-sectional pilot study with the objective of evaluating the prevalence and inter-relationship of cognitive dysfunction, depressive symptoms, and life orientation among patients with chronic kidney disease (CKD) undergoing hemodialysis. The study was conducted at Chandan Hospital, Gomti Nagar, Lucknow, a tertiary care multi-specialty hospital equipped with a specialized nephrology unit. The hospital caters to both urban and rural populations across Uttar Pradesh, ensuring representation of varied socioeconomic and cultural backgrounds. Conducting the study in a single center allowed for methodological consistency, uniform administration of assessment tools, and better control over external variability, thereby enhancing internal validity. The study was carried out over a six-month period, which was considered sufficient for the recruitment of an adequate number of participants for pilot-level analysis.

Ethical considerations

The study protocol was reviewed and approved by the Institutional Ethics Committee (IEC) of Chandan Hospital prior to commencement. All research activities adhered to the principles outlined in the Declaration of Helsinki (2013 revision) and the national ethical guidelines for biomedical research [21]. Written informed consent was obtained from all participants after they were briefed about the study’s objectives, methodology, possible risks, and anticipated benefits. Strict confidentiality was ensured by anonymizing data during analysis. Participation was entirely voluntary, and patients were informed that they could withdraw from the study at any point without any effect on their ongoing medical care.

Participants and sampling

The study population comprised patients diagnosed with CKD and receiving maintenance hemodialysis at the nephrology unit of the hospital. A total of 100 patients were enrolled, representing the maximum number of eligible individuals accessible within the defined six-month study period. Eligibility was determined according to specific inclusion and exclusion criteria. The inclusion criteria required patients to be aged 18 years or older, undergoing hemodialysis for at least six months, and capable both cognitively and physically of completing the assessment tools. Additionally, participants had to provide written informed consent. The exclusion criteria eliminated patients with a history of major psychiatric illness (such as schizophrenia or bipolar disorder), neurological disorders unrelated to CKD (such as stroke, dementia, or epilepsy), severe medical instability, or those unwilling or unable to complete the assessments. Participants were enrolled through a consecutive sampling strategy, whereby all individuals meeting eligibility criteria during the study period were invited to participate. This method maximized representativeness, minimized selection bias, and was operationally feasible within a high-volume clinical setting.

Tools for assessment

To ensure a comprehensive evaluation, three standardized and validated psychometric instruments were employed, supplemented by a structured questionnaire capturing sociodemographic and clinical details.

Montreal Cognitive Assessment (MoCA)

**Figure 1:**
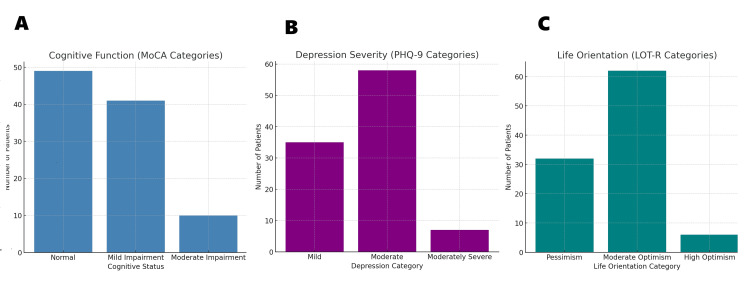
Psychological outcomes of study participants (A) Cognitive function assessed by the MoCA, with nearly half of the participants showing impairment. (B) Depression severity measured using the PHQ-9, showing a high prevalence of moderate depression. (C) Life orientation evaluated by the LOT-R, with most patients reporting moderate optimism and a substantial minority showing pessimism. MoCA: Montreal Cognitive Assessment, PHQ-9: Patient Health Questionnaire-9, LOT-R: Life Orientation Test-Revised

It is a widely recognized screening instrument for identifying mild cognitive impairment (MCI) and assessing diverse cognitive domains, including visuospatial skills, executive functioning, memory, attention, language, abstraction, delayed recall, and orientation [22,23]. The tool has a maximum possible score of 30, with the standard cutoff of 26 commonly used to differentiate normal cognition from cognitive impairment. For this study, scores were categorized into three levels: normal cognition, mild impairment, and moderate impairment. The MoCA was administered in either English or Hindi, based on participant preference, with minor cultural modifications incorporated to enhance contextual appropriateness (Table 1 and Figure 1).

**Table 1:** Distribution of cognitive function (MoCA), depression severity (PHQ-9), and life orientation (LOT-R) scores among hemodialysis patients (N = 100) MoCA: Montreal Cognitive Assessment, PHQ-9: Patient Health Questionnaire-9, LOT-R: Life Orientation Test-Revised, MCI: mild cognitive impairment

Category	Frequency (number)	Percentage (%)
Cognitive function		
Normal cognitive function	49	49
MCI	41	41
Moderate cognitive impairment	10	10
Total	100	100
Patient’s health category		
Mild depression	35	35
Moderate depression	58	58
Moderately severe depression	7	7
Total	100	100
Life orientation category		
Low optimism/high pessimism	32	32
Moderate optimism	62	62
High optimism/low pessimism	6	6
Total	100	100

Patient Health Questionnaire-9 (PHQ-9)

Depressive symptoms were evaluated using the Patient Health Questionnaire-9 (PHQ-9), a standardized instrument comprising nine items aligned with the Diagnostic and Statistical Manual of Mental Disorders IV (DSM-IV) diagnostic criteria for major depressive disorder [24]. Each item is scored on a four-point scale ranging from 0 (“not at all”) to 3 (“nearly every day”), resulting in a cumulative score between 0 and 27. According to established thresholds, scores were classified as mild (5-9), moderate (10-14), moderately severe (15-19), or severe (20-27). The PHQ-9 has been validated for use in Indian populations, supporting its applicability in this study context (Table 1 and Figure 1).

Life Orientation Test-Revised (LOT-R)

Dispositional optimism and pessimism were measured using the LOT-R. The scale comprises 10 items, of which six are scored and four serve as fillers. Responses are recorded on a 5-point Likert scale ranging from “strongly disagree” to “strongly agree.” Based on overall scores, participants were classified into categories of high optimism, moderate optimism, or high pessimism [25]. The LOT-R was chosen for its brevity, reliability, and predictive value in assessing health-related psychological outcomes (Table 1 and Figure 1).

Sociodemographic and Clinical Questionnaire

A structured proforma was used to collect information regarding participants’ age, gender, marital status, family type, educational level, occupation, monthly income, socioeconomic status, residence (urban versus rural), comorbid conditions such as diabetes and hypertension, cause of CKD, and duration on dialysis. This information enabled the exploration of demographic and clinical variations in psychological outcomes.

Data collection procedure

Data were collected before routine dialysis sessions to minimize disruption to patients’ schedules. Each participant was interviewed individually in a private and quiet environment to ensure comfort and reduce distractions. Assessments were administered in a fixed sequence, beginning with the sociodemographic and clinical questionnaire, followed by the MoCA, PHQ-9, and finally the LOT-R [26]. This sequence was chosen to administer the cognitively demanding test (MoCA) first, when participants were most attentive.

Each session lasted approximately 30-40 minutes, with breaks allowed if participants experienced fatigue or discomfort. Investigators administering the tools were trained to maintain neutrality and to avoid influencing responses. In cases where patients scored in the higher ranges of depressive severity or showed signs of significant distress, they were referred to the hospital’s psychiatry department for further evaluation and management. Participation was voluntary, and no financial incentives were offered. However, participants were reassured that their involvement would contribute to improving the understanding of mental health in CKD and potentially inform better clinical practices in the future.

Variables and operational definitions

For the purpose of analysis, dependent variables included cognitive function (measured by MoCA), depressive symptoms (measured by PHQ-9), and life orientation (measured by LOT-R) [27]. The independent variables considered in the study encompassed demographic attributes (age, gender, marital status, educational level, occupation, socioeconomic status, and family type) as well as clinical parameters (etiology of CKD, presence of comorbid conditions, and duration of dialysis treatment). Cognitive function was operationally defined as normal, mild impairment, or moderate impairment based on MoCA scores. Depression severity was defined as mild, moderate, or moderately severe according to PHQ-9 categories. Life orientation was categorized into high optimism, moderate optimism, or high pessimism as per LOT-R scores.

Data management and statistical analysis

All completed questionnaires were checked for accuracy and completeness prior to data entry. The data were coded and analyzed using SPSS version 25 (IBM Corp., Armonk, NY). Descriptive statistics, including frequencies, percentages, means, and standard deviations, were applied to summarize participant characteristics [28]. Associations between categorical variables were examined using the Chi-square test, while independent t-tests were employed to compare continuous variables. Correlation analyses were conducted to explore relationships among MoCA, PHQ-9, and LOT-R scores. A p-value of <0.05 was considered indicative of statistical significance.

Sample size

The final sample size was 100 participants, which reflected the maximum feasible number of patients who met eligibility criteria during the six-month study period. As a pilot study, the aim was not to achieve statistical power for generalizability but to generate preliminary prevalence estimates, identify trends, and assess the feasibility of integrating cognitive, psychological, and psychosocial assessments into dialysis care. The results of this pilot will serve to inform sample size calculations for future multicenter studies.

## Results

**Figure 2:**
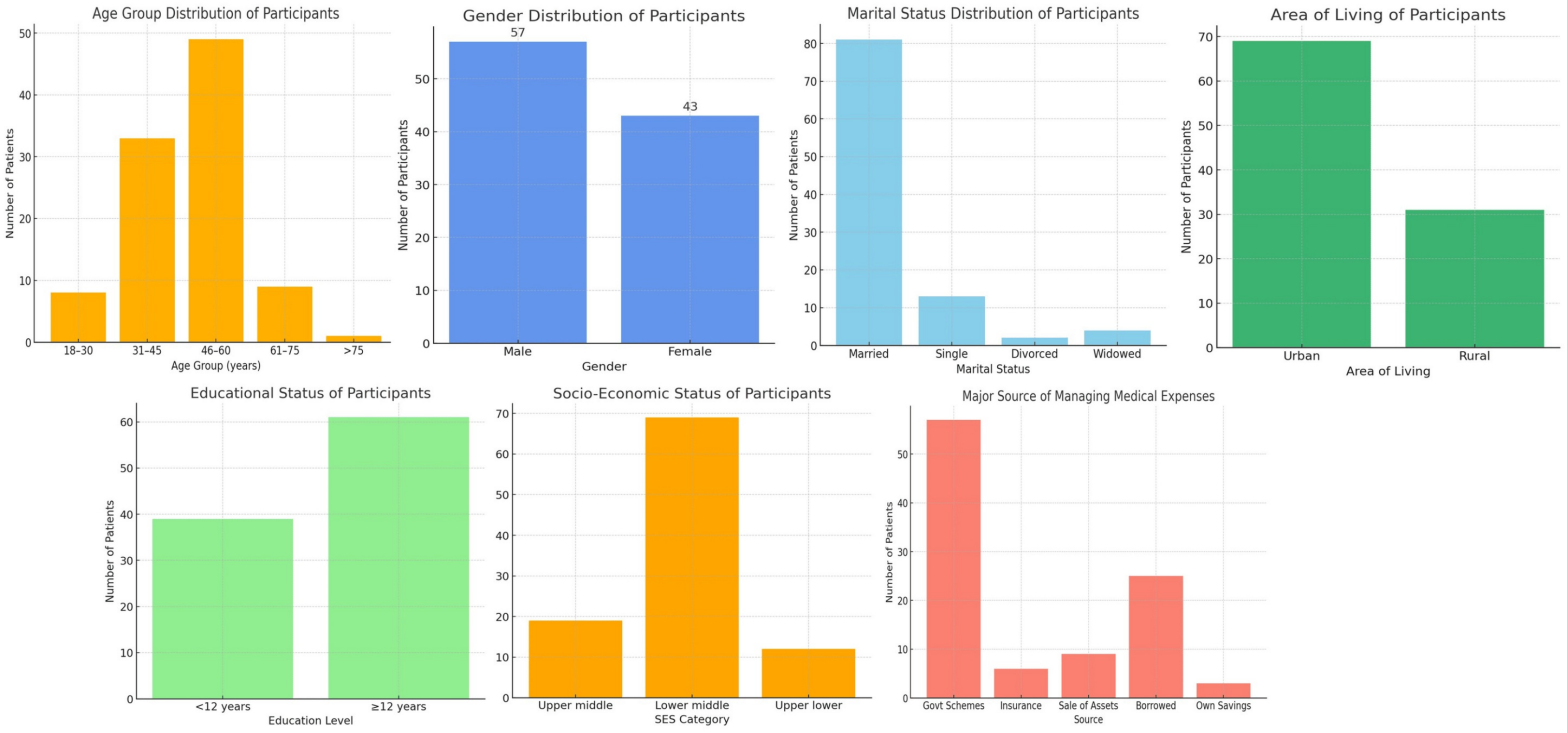
Demographic characteristics of study participants (N = 100) (A) Age group distribution, showing that the majority of patients were aged 46-60 years. (B) Gender distribution, with male participants accounting for 57% and female participants 43%. (C) Marital status distribution, indicating that most participants were married. (D) Area of living, highlighting the predominance of urban residence. (E) Educational status, showing higher education in 61% of patients. (F) Socioeconomic status, with the majority belonging to the lower-middle class. (G) Major source of medical expenses, indicating reliance on government schemes and borrowing.

A total of 100 patients undergoing maintenance hemodialysis were enrolled in this study. The mean age of the cohort was 49.3 ± 12.7 years, with the majority belonging to the 46-60 years age group (49%), followed by 31-45 years (33%). Only 1% were above 75 years of age. Male participants constituted 57% of the study population, while female participants accounted for 43%. Sociodemographic analysis revealed that most patients were married (81%), lived in nuclear families (62%), and came predominantly from urban areas (69%). With respect to education, 61% had completed more than 12 years of schooling, while 39% had lower levels of education. The socioeconomic distribution indicated that 69% belonged to the lower-middle class, 19% to the upper-middle class, and 12% to the upper-lower category (Figure 2).

Cognitive function

Cognitive status, assessed using the MoCA, showed that 49% of patients had normal cognitive function, while 41% demonstrated mild cognitive impairment and 10% exhibited moderate impairment (Table 2). Cognitive dysfunction was more prevalent among older patients and those with lower educational attainment. When stratified by education, 74% of participants with less than 12 years of education exhibited impairment, compared to only 39% of those with higher education. This difference was statistically significant (χ² = 8.92, p < 0.01).

**Table 2:** Sociodemographic and socioeconomic characteristics of study participants (N = 100)

Category	Frequency (number)	Percentage (%)
Age group		
18-30 years	8	8
31-45 years	33	33
46-60 years	49	49
61-75 years	9	9
Above 75 years	1	1
Total	100	100
Gender		
Male	57	57
Female	43	43
Total	100	100
Marital status		
Married	81	81
Single	13	13
Divorced	2	2
Widowed/widower	4	4
Total	100	100
Number of children		
None	15	15
1	14	14
2	42	42
More than 2	29	29
Total	100	100
Area of living		
Urban area	69	69
Rural area	31	31
Total	100	100
Religion		
Hinduism	59	59
Islam	37	37
Christianity	2	2
Sikhism	2	2
Total	100	100
Type of family		
Nuclear	62	62
Joint	33	33
Extended	5	5
Total	100	100
Working status		
Currently working	58	58
Not working	33	33
Retired	5	5
Homemaker	4	4
Total	100	100
Educational status		
Less than 12 years of education	39	39
More than 12 years of education	61	61
Total	100	100
Educational qualification of the head of the family		
Primary school	4	4
Middle school	15	15
High school	14	14
Intermediate diploma	33	33
Graduation	26	26
Professional degree	8	8
Total	100	100
Occupation of the head of the family		
Unemployed	1	1
Elementary occupation	6	6
Plant and machine operators and assemblers	10	10
Craft and related trade workers	22	22
Skilled agricultural and fishery workers	28	28
Skilled workers, shop and market sales workers	19	19
Clerk	6	6
Technicians/associate professionals	5	5
Professional	3	3
Total	100	100
Monthly income of the family		
7,316-21,913	12	12
21,914-36,526	27	27
36,527-45,588	24	24
45,589-54,650	17	17
54,651-59,251	20	20
Total	100	100
Socioeconomic status		
Upper middle	19	19
Lower middle	69	69
Upper lower	12	12
Total	100	100
Medical expenses funding agency		
Government schemes/funds (CM FUND/AYUHMAN)	57	57
Health insurance	6	6
Sale of assets	9	9
Borrow from friends/family	25	25
Own savings	3	3
Total	100	100

Depressive symptoms

Patient’s health status based on depressive symptoms, as measured by the PHQ-9, reported that there was a high prevalence of depressive symptoms, where 58% of patients reported moderate depression, 7% reported moderately severe depression, and 35% had mild depression (Table 2). No cases of severe depression were observed. Gender differences were notable, with men more likely to report moderate or moderately severe depression (70%) compared to women (58%). Although not statistically significant, the trend suggested a higher psychological burden among male dialysis patients.

Life orientation

Analysis of the life orientation revealed that 32% of participants had a pessimistic outlook, 62% demonstrated moderate optimism, and only 6% exhibited high optimism (Table 2). Patients with lower socioeconomic status and limited education were more likely to score in the pessimistic category, suggesting the influence of contextual factors such as financial stress and restricted coping resources.

Clinical characteristics

**Figure 3:**
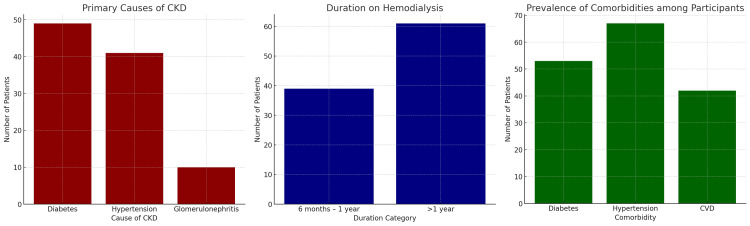
Clinical characteristics of study participants (N = 100) (A) Primary causes of CKD, showing diabetes and hypertension as the leading etiologies, followed by glomerulonephritis. (B) Duration on hemodialysis, with the majority of participants receiving dialysis for more than one year. (C) Prevalence of comorbid conditions, indicating high rates of hypertension, diabetes, and cardiovascular disease. CKD: chronic kidney disease, CVD: cardiovascular disease

Diabetes and hypertension were the leading causes of CKD in this cohort, accounting for 49% and 41% of cases, respectively, while glomerulonephritis was implicated in 10% (Table 2). The prevalence of comorbidities was high, with 53% of patients reporting diabetes, 67% hypertension, and 42% cardiovascular disease. Regarding dialysis duration, 61% of participants had been on dialysis for more than one year, while 39% had a shorter history (Table 2). Longer dialysis duration was associated with greater cognitive impairment, although this did not reach statistical significance (Figure 3).

Cross-tabulation of key findings

Cross-tabulation analyses highlighted several important patterns. Cognitive impairment was significantly more common among patients with lower educational levels, as noted earlier. Gender-based comparisons revealed that male patients were more likely to experience moderate to severe depression (70% versus 58% in female patients). Similarly, pessimistic life orientation was disproportionately represented among participants with low socioeconomic status, with 58% of upper- to lower-class patients scoring in the pessimism range compared to only 18% of upper- to middle-class participants.

Table 2 summarizes the distribution of age, gender, marital status, number of children, area of living, religion, family type, employment status, education level, education and occupation of the head of family, monthly family income, socioeconomic class, and major sources of managing medical expenses.

Correlation analysis

A Pearson correlation analysis was conducted to evaluate associations between cognitive function (MoCA scores), depression severity (PHQ-9 scores), and life orientation (LOT-R scores) among patients with CKD. Results revealed a significant negative correlation between MoCA and PHQ-9 scores (r = -0.42, p < 0.001), indicating that lower cognitive scores were associated with higher levels of depression. Additionally, a significant positive correlation was observed between MoCA and LOT-R scores (r = 0.36, p = 0.001), suggesting that higher optimism was related to better cognitive status. PHQ-9 scores were negatively correlated with LOT-R scores (r = -0.48, p < 0.001), indicating that individuals with greater depressive symptoms had lower optimism levels (Table 3). These findings highlight the interlinked nature of psychological and cognitive functioning in patients with CKD.

**Table 3:** Correlation between cognitive function, patient health status, and life orientation **p < 0.001 MoCA: Montreal Cognitive Assessment, PHQ-9: Patient Health Questionnaire-9, LOT-R: Life Orientation Test-Revised

Variables	MoCA	PHQ-9	LOT-R
MoCA	1	-0.42**	0.36**
PHQ-9	-0.42**	1	-0.48**
LOT-R	0.36**	-0.48**	1

## Discussion

The findings of this pilot study underscore the high prevalence of cognitive dysfunction, depressive symptoms, and pessimistic life orientation among patients with CKD undergoing hemodialysis [28]. More than half of the participants exhibited measurable cognitive deficits, while nearly two-thirds had clinically relevant depression. Only a small fraction of patients demonstrated high optimism, with pessimistic orientations particularly common among socioeconomically disadvantaged groups. These results highlight critical gaps in the recognition and management of psychological and cognitive health within nephrology practice in India.

Cognitive dysfunction in dialysis patients

Our results, which found that 51% of patients experienced some degree of cognitive impairment, are consistent with international reports documenting prevalence rates of 30%-80% among dialysis populations. For example, a German cohort study reported that up to 75% of hemodialysis patients demonstrated measurable cognitive decline [29]. Mechanistically, CKD predisposes patients to cognitive dysfunction through uremic toxin accumulation, microvascular injury, chronic inflammation, and repeated cerebral hypoperfusion during dialysis [30]. The finding that lower education was strongly associated with impairment supports the concept of cognitive reserve, where higher educational attainment provides protective buffering against neuropathological burden [31].

Depression and its overlap with CKD symptoms

Depression was strikingly prevalent in this cohort, with 65% of participants exhibiting moderate or higher severity. These figures exceed global estimates of 20%-40% but are comparable to reports from other low- and middle-income countries where psychosocial stressors and limited access to mental health care exacerbate burden [32]. Notably, depressive symptoms overlap with common CKD manifestations such as fatigue, anorexia, and sleep disturbances, leading to frequent under-recognition by nephrologists [33]. Our findings reinforce the need for the routine use of simple screening instruments, such as the PHQ-9, in dialysis units. The observed gender differences, with men more frequently reporting moderate to severe depression, diverge from global trends where women typically exhibit higher prevalence. Cultural factors, gender roles, and economic pressures on men in the Indian context may partially explain this discrepancy [34].

Life orientation as a mediator

The analysis of life orientation provides novel insights into the psychosocial landscape of patients with CKD. A third of participants reported pessimistic outlooks, while high optimism was rare. Optimism was positively correlated with cognitive performance and inversely associated with depression, suggesting a protective role. This supports findings from studies in cardiovascular and oncology patients, where optimism has been linked with better treatment adherence, reduced psychological distress, and improved long-term outcomes [35]. In the dialysis context, pessimism may exacerbate the emotional toll of chronic illness, while optimism may serve as a resilience factor. Despite its importance, life orientation is rarely assessed in nephrology practice. Incorporating this dimension into psychological evaluations could provide clinicians with valuable insights into patients’ coping styles and risk of poor adjustment.

Interrelationship of cognitive dysfunction, depression, and pessimism

The observed associations highlight the intricate relationship between cognitive function and emotional well-being in patients with CKD. Depression was inversely correlated with cognitive performance, consistent with the concept of pseudodementia, wherein mood disturbances can imitate or aggravate cognitive deficits. Conversely, a higher degree of optimism appeared to provide a protective effect against both cognitive impairment and depressive symptoms. These results suggest that interventions targeting depressive symptoms may confer concomitant cognitive benefits, while fostering optimism through psychosocial strategies could enhance resilience and overall psychological health [36].

Diagnostic gaps and clinical implications

Despite the high prevalence of impairment, none of the patients in this study had documented prior assessments for cognition, depression, or life orientation. This diagnostic gap is reflective of broader clinical practice in resource-constrained settings, where the primary focus remains on physical aspects of CKD. However, unrecognized cognitive dysfunction compromises treatment adherence, while untreated depression contributes to poor quality of life, higher hospitalization rates, and mortality. The findings advocate strongly for integrating routine psychological screening into nephrology care, particularly using brief, validated instruments such as MoCA, PHQ-9, and LOT-R [37]. Cognitive decline, frequently accompanied by anxiety and depression, is common in patients with CKD. Regular physical activity has been shown to mitigate both cognitive dysfunction and depressive symptoms across all disease stages [38]. Studies suggest that practicing yoga and meditation helps alleviate stress and anxiety, leading to better health outcomes and enhanced quality of life in individuals with CKD [39].

## Conclusions

This preliminary study sheds light on the significant psychological hurdles faced by individuals with chronic kidney disease who are undergoing hemodialysis. The frequent co-occurrence of cognitive impairment, depression, and pessimism underscores the complex interplay between physical and mental health in this population. The results suggest that these psychological issues are not only prevalent but also interconnected, with important consequences for patient outcomes and quality of life. The lack of prior screening for these conditions highlights a major gap in current nephrology care practices, emphasizing the need for a more thorough approach to patient evaluation and management.

The study’s suggestions for regular screening using brief assessment tools and the inclusion of psychosocial support represent a crucial change in the management of chronic kidney disease. By addressing the psychological aspects of the disease along with physical symptoms, healthcare providers can potentially improve overall patient outcomes and well-being. The identified connection between cognition, depression, and life orientation further supports the necessity for comprehensive care. By demonstrating the interplay of cognitive and emotional health in CKD, this pilot study lays the groundwork for future research and reinforces the importance of integrating mental health into holistic CKD management.

## Appendices

Research informed consent is provided in Table 4. 

**Table 4:** Research informed consent

Title of the study	Cognitive Dysfunction and Life Orientation Among Chronic Kidney Disease Patients: An Interventional Study
Primary researcher	Name: Shikha, Department: Human Development and Family Studies, Babasaheb Bhimrao Ambedkar University, Vidya-Vihar, Raebareli Road, Lucknow, Uttar Pradesh, Email ID: researchscholarshikhag@gmail.com
Purpose of the study	To determine the socioeconomic status of the respondents, to assess life orientation and patients’ health status based on depressive symptoms of patients with chronic kidney disease, and to assess the cognitive function of patients with chronic kidney disease
Procedure	You will be asked a few questions, and you have to answer them as honestly as you can.
Risks	There is no physical or mental, intentional or unintentional harm, or any discomfort involved in this research.
Benefits	Cognitive dysfunction is associated with poor quality of life and may lead to difficulties in the effective treatment of the primary disease. Early diagnosis and identification of influencing factors, along with timely intervention, can help the treating team improve patients’ physical, mental, and social well-being.
Confidentiality	Please do not write any identifying information. Every effort will be made by the researcher to preserve your confidentiality, including assigning code names/numbers for participants to be used on all research notes and documents, and keeping notes, interview transcriptions, and other identifying information in a locked file cabinet in the researcher’s possession. Participant data will be kept confidential, except in cases where the researcher is legally obligated to report incidents such as abuse or suicide risk.
Contact information	For questions or concerns about this study or if you experience any adverse effects, contact: Primary Researcher: Shikha, Email: researchscholarshikhag@gmail.com. For questions regarding your rights as a research participant, contact the researcher using the above information.
Voluntary participation	Participation in this study is voluntary. You may decide whether or not to take part. Even after signing the consent form, you are free to withdraw at any time without giving a reason. Withdrawing will not affect your relationship with the researcher. If you withdraw before data collection is completed, your data will be returned to you or destroyed.
Consent statement	I have read and understood the provided information and have had the opportunity to ask questions. I understand that participation is voluntary and that I am free to withdraw at any time, without giving a reason and without cost. I understand that I will be given a copy of this consent form. I voluntarily agree to take part in this study.
Signatures	Participant’s signature: ________________________ Date: _______________ Researcher’s signature: ________________________ Date: _______________

## References

[1] Liu W, Zhou L, Yin W, Wang J, Zuo X (2023). Global, regional, and national burden of chronic kidney disease attributable to high sodium intake from 1990 to 2019. Front Nutr.

[2] Standl E, Khunti K, Hansen TB, Schnell O (2019). The global epidemics of diabetes in the 21st century: current situation and perspectives. Eur J Prev Cardiol.

[3] Talukdar R, Ajayan R, Gupta S (2025). Chronic kidney disease prevalence in India: a systematic review and meta-analysis from community-based representative evidence between 2011 to 2023. Nephrology (Carlton).

[4] Bhujbal R, Ram A (2021). Prevalence of chronic kidney disease and hemodialysis related problems for patients in India. Int Res J Eng Tech.

[5] Kushwaha R, Vardhan PS, Kushwaha PP (2023). Chronic kidney disease interplay with comorbidities and carbohydrate metabolism: a review. Life (Basel).

[6] Pereira AA, Weiner DE, Scott T, Sarnak MJ (2005). Cognitive function in dialysis patients. Am J Kidney Dis.

[7] Andrews TD, Day GS, Irani SR, Kanekiyo T, Hickson LJ (2025). Uremic toxins, CKD, and cognitive dysfunction. J Am Soc Nephrol.

[8] Qi Z, Chu X, Li S (2026). Macelignan, a lignan from Myristica fragrans Houtt., rescues mitochondrial homeostasis and prevents cognitive decline in vascular dementia by modulating the mTOR-Mitophagy axis. J Ethnopharmacol.

[9] (2023). Neurocritical Care Nursing Management of Stroke, An Issue of Critical Care Nursing Clinics of North America. Stroke, An Issue of Critical Care Nursing Clinics of.

[10] Pépin M, Giannakou K, Levassort H (2025). Care pathways for patients with cognitive impairment and chronic kidney disease. Nephrol Dial Transplant.

[11] Lu Y, Zhai S, Liu Q, Dai C, Liu S, Shang Y, Chen C (2024). Correlates of symptom burden in renal dialysis patients: a systematic review and meta-analysis. Ren Fail.

[12] Sharp J, Brown JS (2024). The intersection of chronic kidney disease and depression. Nephrol Nurs J.

[13] Ogieuhi IJ, Aderinto N, Olatunji G (2025). The socioeconomic impact of kidney disease on African families: a scoping review. Discov Public Health.

[14] Ong SC, Butt MD, Malik T (2025). Depression in kidney failure patients. Handbook of the Behavior and Psychology of Disease.

[15] Fairbank EJ, Borenstein-Laurie J, Alberts NM, Wrosch C (2024). Optimism, pessimism, and physical health among youth: a scoping review. J Pediatr Psychol.

[16] Saedi F, Dehghan M, Mohammadrafie N, Xu X, Hermis AH, Zakeri MA (2024). Predictive role of spiritual health, resilience, and mental well-being in treatment adherence among hemodialysis patients. BMC Nephrol.

[17] Mouta S, Fonseca Vaz I, Pires M, Ramos S, Figueiredo D (2023). What do we know about pseudodementia?. Gen Psychiatr.

[18] Febria F, Kusumiati RY (2025). Optimism as a predictor of resilience in aging: counseling approaches and interventions. J Bimbingan Konseling Terap.

[19] Bolignano D, Simeoni M, Hafez G (2025). Cognitive impairment in CKD patients: a guidance document by the CONNECT network. Clin Kidney J.

[20] Ulasi I, Sumaili EK, Bajpai D, Claure-Del Granado R, Hughes JT (2025). Regional perspectives of inequities in chronic kidney disease: beyond the obvious. Adv Kidney Dis Health.

[21] (2022). Research must do no harm: new guidance addresses all studies relating to people. Nature.

[22] Nasreddine ZS, Phillips NA, Bédirian V (2005). The Montreal Cognitive Assessment, MoCA: a brief screening tool for mild cognitive impairment. J Am Geriatr Soc.

[23] Islam N, Hashem R, Gad M (2023). Accuracy of the Montreal Cognitive Assessment tool for detecting mild cognitive impairment: a systematic review and meta-analysis. Alzheimers Dement.

[24] Kroenke K, Spitzer RL, Williams JB (2001). The PHQ-9: validity of a brief depression severity measure. J Gen Intern Med.

[25] Scheier MF, Carver CS, Bridges MW (1994). Distinguishing optimism from neuroticism (and trait anxiety, self-mastery, and self-esteem): a reevaluation of the Life Orientation Test. J Pers Soc Psychol.

[26] Bazo-Alvarez JC, Aparicio AR, Robles-Mariños R, Julca-Guerrero F, Gómez H, Bazo-Alvarez O, Cjuno J (2024). Cultural adaptation to Bolivian Quechua and psychometric analysis of the Patient Health Questionnaire PHQ-9. BMC Public Health.

[27] Rotenberg S, Anderson ND, Binns MA (2024). Effectiveness of a meta-cognitive group intervention for older adults with subjective cognitive decline or mild cognitive impairment: the ASPIRE randomized controlled trial. J Prev Alzheimers Dis.

[28] Alabi O, Bukola T (2023). Introduction to descriptive statistics. Recent Advances in Biostatistics.

[29] Asfour NM, Almutairi AM (2024). The relationship between reactive depression and kidney failure patients. Int J Neurol Sci.

[30] Golenia A, Zolek N, Olejnik P, Wojtaszek E, Glogowski T, Malyszko J (2023). Prevalence of cognitive impairment in peritoneal dialysis patients and associated factors. Kidney Blood Press Res.

[31] McIntyre CW, Jain A (2025). Dialysis and cognitive impairment. Nat Rev Nephrol.

[32] Elkana O, Beheshti I (2024). Education as a proxy for cognitive reserve: moderating effects on white matter hyperintensity burden in healthy aging and cognitive decline. medRxiv.

[33] Yu R, Perera C, Sharma M (2023). Child and adolescent mental health and psychosocial support interventions: an evidence and gap map of low- and middle-income countries. Campbell Syst Rev.

[34] Țenea-Cojan ȘT, Dinescu VC, Gheorman V (2025). Exploring multidisciplinary approaches to comorbid psychiatric and medical disorders: a scoping review. Life (Basel).

[35] Kupper N, Post N, Kop WJ, Widdershoven J (2025). The longitudinal association of optimism with quality of life after percutaneous coronary intervention for coronary heart disease; the THORESCI study. Gen Hosp Psychiatry.

[36] Reivich K, Gillham JE, Chaplin TM, Seligman ME (2013). From helplessness to optimism: the role of resilience in treating and preventing depression in youth. Handbook of Resilience in Children.

[37] Sagala IM, Rifai A, Satiti IA, Setyabudhi V (2025). Validity and reliability of MoCA-Ina for assessing cognitive function in dialysis patients with chronic kidney disease. CRJIM.

[38] Shikha Shikha, Kiran UV (2023). Combating cognitive dysfunction among CKD patients: need for effective treatment module. J Reatt Ther Dev Divers.

[39] Gautam S, Kiran UV (2024). Clinical effects of yoga and meditational practices on the holistic health of chronic kidney disease patients: a systematic review. Cureus.

